# Optical coherence tomography confirms non‐malignant pigmented lesions in phacomatosis pigmentokeratotica using a support vector machine learning algorithm

**DOI:** 10.1111/srt.13377

**Published:** 2023-05-30

**Authors:** Jenna Lee, Mohammad Javad Beirami, Reza Ebrahimpour, Carolina Puyana, Maria Tsoukas, Kamran Avanaki

**Affiliations:** ^1^ Department of Dermatology University of Illinois‐Chicago Chicago Illinois USA; ^2^ Center for Cognitive Science Institute for Convergence Science and Technology (ICST) Sharif University of Technology Tehran Islamic Republic of Iran; ^3^ Department of Computer Engineering Shahid Rajaee Teacher Training University Tehran Islamic Republic of Iran; ^4^ School of Cognitive Sciences Institute for Research in Fundamental Sciences (IPM) Tehran Islamic Republic of Iran; ^5^ Department of Biomedical Engineering University of Illinois‐Chicago Chicago Illinois USA

**Keywords:** attenuation coefficient, atypical nevus, dysplastic nevus, Gabor wavelet transformation, machine learning, nevus spilus, optical coherence tomography, phacomatosis pigmentokeratotica, skin cancer, Spitz nevus, support vector machine

## Abstract

**Introduction:**

Phacomatosis pigmentokeratotica (PPK), an epidermal nevus syndrome, is characterized by the coexistence of nevus spilus and nevus sebaceus. Within the nevus spilus, an extensive range of atypical nevi of different morphologies may manifest. Pigmented lesions may fulfill the ABCDE criteria for melanoma, which may prompt a physician to perform a full‐thickness biopsy.

**Motivation:**

Excisions result in pain, mental distress, and physical disfigurement. For patients with a significant number of nevi with morphologic atypia, it may not be physically feasible to biopsy a large number of lesions. Optical coherence tomography (OCT) is a non‐invasive imaging modality that may be used to visualize non‐melanoma and melanoma skin cancers.

**Materials and Method:**

In this study, we used OCT to image pigmented lesions with morphologic atypia in a patient with PPK and assessed their quantitative optical properties compared to OCT cases of melanoma. We implement a support vector machine learning algorithm with Gabor wavelet transformation algorithm during post‐image processing to extract optical properties and calculate attenuation coefficients.

**Results:**

The algorithm was trained and tested to extract and classify textural data.

**Conclusion:**

We conclude that implementing this post‐imaging machine learning algorithm to OCT images of pigmented lesions in PPK has been able to successfully confirm benign optical properties. Additionally, we identified remarkable differences in attenuation coefficient values and tissue optical characteristics, further defining separating benign features of pigmented lesions in PPK from malignant features.

**Abbreviations**
AC: attenuation coefficient
DEJ: dermal–epidermal junction
OCT: optical coherence tomography
PPK: Phacomatosis pigmentokeratotica
ROI: region of interest
SLN: speckled lentiginous nevus
SVM: support vector machine

## 1 INTRODUCTION

Phacomatosis pigmentokeratotica (PPK) is a distinct and rare type of epidermal nevus syndrome characterized by coexisting speckled lentiginous nevus (SLN) of the papular type and nonepidermolytic organoid sebaceous nevus.^1^ Patients with PPK also present with extracutaneous symptoms, which may include neurological, musculoskeletal, and ocular disorders, commonly correlating to the limbs affected cutaneously.^1^, ^2^, ^3^ A systematic search retrieved 95 cases reported in literature.^4^ PPK is hypothesized to be due to a single dominant heterozygous activating HRAS c.37G>A mutation, which causes the two different types of nevi. The mutation affects a multi‐potent progenitor cells, which then gives rise to cutaneous and extracutaneous manifestations seen in PPK. Sebaceous nevus, otherwise known as nevus sebaceous of Jadassohn, is a congenital malformation that involves hamartomas of the pilosebaceous follicular unit. The coexisting SLN, otherwise known as nevus spilus, is described as larger café‐au‐lait macules with numerous nevi or smaller superimposed darker black or brown melanocytic proliferations.^5^ Sizes of the nevi may range from a millimeter up to 10 cm.^2^, ^5^, ^6^ Spitz nevi may also be found within speckled lentiginous nevi of PPK patients.^7^, ^8^, ^9^, ^10^ Within regions of the SLN, secondary cutaneous manifestations are rare; however, cases of malignant melanoma have been reported.^3^, ^11^ Atypical nevi, otherwise known as dysplastic nevi, are melanocytic neoplasms with clinical features that may simulate melanoma (topographical asymmetry, color variegation, large diameter [>6 mm]).^12^ Patients with PPK may have atypical nevi that may be difficult to discriminate from melanoma due to morphologic atypia. Often times, patients with PPK or other nevus syndromes are subject to a significant number of biopsies. Patients with PPK may have a high propensity of developing nevi with atypia, which can fulfill the “ABCDE” criteria for melanoma.

PPK is a clinical diagnosis involving the identification of characteristic symptoms of an epidermal nevus syndrome, comprehensive patient history, and thorough physical examination.^2^ Additional testing, such as full skeletal, should be performed. Routine central nervous system (CNS) examinations are not standard unless the patient presents with developmental concerns, CNS symptoms, or if the epidermal nevus is largely present within the craniofacial distribution.^2^ Treatment of PPK is primarily reserved for extracutaneous involvement, such as limb length discrepancy, seizures, or ocular manifestations, while surgical excision may be used to address symptomatic nevi and nevi with clinically worrisome morphologic atypia.

Generally, for melanocytic lesions, the gold standard for a clinical suspicion of melanoma is a full‐thickness biopsy of the lesion, which allows for adequate histopathologic interpretation and determination of margins of resection.^13^ Atypical nevi can often be asymmetric, have irregular borders, different colors, diameters >6 mm, and evolve over time, fulfilling clinical diagnostic criteria for suspicion of melanoma.^14^ Moreover, visual inspection only has a specificity of 59%−78% and is highly dependent on physician expertise.^15^ Approximately 15−30 benign lesions are biopsied to diagnose one melanoma.^16^ Biopsies result in significant pain, scarring, mental distress, and disfigurement to the patient. These factors are significantly increased in patients with numerous atypical nevi or nevus syndromes, such as PPK. Numerous non‐invasive imaging technologies have been developed; however, they lack diagnostic specificity and accuracy to differentiate melanoma from benign nevi. The current literature involving PPK includes case reports and studies on genetics, but none explores the pigmented lesions within PPK. In this study, we investigated the utility of optical coherence tomography (OCT) imaging of atypical nevi in a patient with PPK to confirm non‐malignant features with the goal of preventing unnecessary biopsy.

OCT is an emerging non‐invasive imaging technology that generates cross‐sectional images of a tissue in real time.^17^, ^18^, ^19^, ^20^ It uses a near‐infrared low coherence light source^21^ and has imaging capability of up to 2 mm in depth and up to 6 mm in width.^22^ Swept‐source OCT has a high spatial resolution of less than 10 μm, which is 10−100 times finer than clinical high‐frequency ultrasound.^23^ Optical imaging is based on the concept of light as electromagnetic waves with different wavelengths and intensities. Light wave energy levels have unique capabilities of interacting with different tissue components and microstructures based on their inherent tissue optical properties.^24^ Light–tissue interaction is due to diffuse scattering, specular scattering, and absorption of light. Diffuse scattering is caused by incident photons scattering at different refractive indices due to biological compartments in the tissue. Specular scattering is due to light being reflected at the same incident angle compared to normal light. Absorption of light is caused by biological chromophores and fluorophores within tissue structures.^23^, ^24^, ^25^ Both scattering and absorption of light affect light reflectance and attenuation. Other methods utilizing light–tissue interactions have been developed to diagnose skin diseases. Full‐field OCT (FF‐OCT) uses wide‐field illumination rather than beam scanning.^26^ Line‐field confocal OCT (LC‐OCT) uses a broadband laser coupled with line detection using a line‐scan camera where the focus is continuously adjusted during the scan to achieve confocal spatial filtering.^27^ Reflectance confocal microscopy (RCM) is another method for high‐resolution skin imaging for diagnostic purposes. RCM also uses confocal illumination to display high‐resolution images based on changes in the refractive index of tissue, but its penetration depth is limited to approximately 200–250 μm.^28^

Melanin has a high absorption in both broad spectrum visible light and near‐infrared light bands.^29^ Based on light–tissue interaction theories, pleomorphic malignant cells are altered biological tissue and thus will have differences in refractive index and absorptive properties compared to normal cells. This indicates that OCT should discriminate benign from malignant lesions.^30^, ^31^, ^32^, ^33^, ^34^, ^35^, ^36^, ^37^, ^38^, ^39^ However, swept‐source OCT has a specificity of only about 61% when detecting melanoma.^40^ LC‐OCT, with its confocal capabilities, has demonstrated success at identifying melanocytic lesions with higher accuracy.^41^ RCM has also demonstrated adequate sensitivity (93.5%) and specificity (78.8%) for melanoma diagnosis,^42^ but the device has mostly been implemented in large hospitals and academic and research centers.^43^ FF‐OCT acquires images en face, and while it has been used to identify different skin tumors, it does not appear to have been applied to identification of melanoma in situ (MIS).^44^

The goal of this study is to investigate the ability of swept‐source OCT to detect malignancy within pigmented lesions of PPK. This is done via post‐image processing on MATLAB and machine learning. We apply a computer‐based analysis to the OCT image, which is essential to analyze large quantities of data. This allows for the illumination of anatomical and functional features of the lesion to a greater degree than the human eye. The application of the algorithm to the OCT image extracts quantitative properties of the skin, such as attenuation coefficient (AC) and textural data, thereby differentiating unique benign optical properties of pigmented lesions in PPK from melanoma.

With the addition of computer‐based analysis coupled with this non‐invasive imaging technique and through understanding ACs, we aim to investigate the utility of OCT confirmation of benign etiology of pigmented lesions, without the physical, cosmetic, time, and financial repercussions of a biopsy. With modern technological advances such as artificial intelligence in skin disease identification, understanding non‐invasive biomarker optical features is necessary to identify characteristics of this disease. Application of the results of this study may then be used to train a deep learning network and further artificial intelligence applications to identify benign and malignant features of skin pathologies.

## 2 MATERIALS AND METHODS

All imaging procedures and experimental protocols were approved and carried out based on guidelines of the Institutional Review Board of University of Illinois at Chicago College of Medicine (IRB #2021‐0249). Informed consent was obtained from all subjects prior to enrollment in the study.

### 2.1 Patient population

Benign nevi with morphological atypia were imaged from a 30‐year‐old female patient with history significant for PPK. She presented with a sebaceous nevus on her right lateral neck, which extended midline anteriorly and chest distally. She had numerous SLN of the papulosa variant, which presented in a checkerboard pattern involving both sides of the face, left and predominantly right shoulders, right upper back, right upper chest, lower back, and right lower extremity. Within areas of SLN are a significant number of typical and atypical nevi. Past excised lesions include numerous pigmented lesions of benign morphology, nevus sebaceous with syringocystadenoma papilliform, and trichoblastoma. Previous pathology reports dated from the patient's birth to the present were analyzed. The patient had significant history and previous biopsies of pigmented lesions including compound dysplastic nevi and compound Spitz nevi. She has numerous similar morphological manifestations of Spitz nevi, which were described as occasionally pruritic, pink, dome shaped, oval nevi with central blue–white veil globules. This patient was selected to be imaged due to her wide variety of nevi of atypical morphology and type, without history or family history of melanoma or skin cancer.

For atypical benign nevi in our PPK patient, inclusion criteria for this study were as follows: pigmented lesions ≥3 mm in width and/or length, >0 mm in vertical height or raised, textural changes, irregular borders, atypical color patterns. Pigmented lesions that fulfilled all of the following categories were included: <3 mm diameter, macular, symmetrical, and regular borders. Selected PPK‐atypical nevi are likely compound spitz nevi or compound dysplastic nevi and have not demonstrated changes to the lesion within the last 10 years. An image was taken with an iPhone XS (Apple Inc.), with a dermoscopy attachment (DermLite DL4N) in a polarized light setting with 90% isopropyl alcohol as a medium. Examples of selected nevi seen in Figure 5. Separate sets of nevi were used for training the model and testing the model. Biopsies were not performed on the nevi from the PPK patient used for analysis in order to avoid trauma to the PPK patient. This was appropriate because the patient has been under expert dermatologic care for more than 25 years including careful monitoring of all atypical nevi for changes in appearance that could indicate the presence of melanoma, and the family has no history of melanoma or skin cancer.

Patients with melanoma were identified in the clinic. A suspected pigmented lesion, which fulfilled “ABCDE” requirements of melanoma, was imaged with OCT prior to full‐thickness wide excision. Patients with histopathologically proven melanoma were included in this study. Selected patients for this study included a 59‐year‐old male with MIS the lower leg, a 43‐year‐old female with MIS on the leg, a 50‐year‐old male with MIS on his lower leg, and a 59‐year‐old female with superficial spreading melanoma on the head with Breslow depth 0, 48 mm in size, with one mitosis.

### 2.2 OCT configuration

The OCT used in this study was a multi‐beam, swept‐source system (Vivosight, Michelson Diagnostic Inc.) with a hand‐held probe used for skin imaging. The light source consisted of a broadband laser with a central wavelength of 1305 ± 15 nm. The scanning area of the OCT measured 6 mm in width × 6 mm in length × 2 mm in depth. It had an axial resolution of 10 μm and a lateral resolution of 7.5 μm. The OCT image is created by the reflectivity profile or the change in reflectivity with depth. This reflectivity profile is called the axial scan (A‐line or A‐scan). To generate a cross‐sectional image, or the B‐scan, the OCT system combines several A‐lines for each transverse position of an incident beam on the biological tissue structure.^45^ All suspected melanoma lesions, as well as all PPK nevi were identified and cleaned with an alcohol prep pad. Next, the OCT probe was applied perpendicular to the skin. A total of 120 B‐scan en face images were collected per lesion of interest.

### 2.3 Computational algorithms

#### 2.3.1 Calculation of attenuation coefficient and analysis

When light waves penetrate tissue, the intensity decays exponentially due to light scattering and absorption of the tissue microstructures under different physiological conditions.^46^ This attenuation of light is quantified by the AC and is governed by Beer–Lambert law.^47^ In literature, the study of ACs has proven to be successful in characterizing tissue and structural changes.^46^, ^48^, ^49^ In this study, a method is used to calculate ACs by converting each pixel of region of interest (ROI) of an OCT image and converting it to a corresponding “optical absorption coefficient pixel.” This method allows for improved accuracy in detecting data in homogenous and heterogeneous tissue without pre‐segmenting or pre‐averaging.^24^, ^46^ The single scattering equation implemented is as: *I*(*x*) = *I*_0_ *ρ*e^−2^ *^μx^*, where *I* is the value of detected intensity, *I*_0_ is the incident light intensity, *ρ* is the backscattering coefficient, *μ* is AC, and *x* is the depth. The factor, 2, accounts for the light traveling to the tissue and back to detector. The calculation of AC is done by fitting an exponential curve to the above equation, from which a decay constant can be extracted. An ROI must be selected within a depth range before fitting the curve.^47^ The extracted values are then averaged, smoothed, and fitted to a polynomial equation. The resultant slope of the equation yields the AC of the ROI.^24^

#### 2.3.2 Image processing for skin surface detection

When imaging skin on OCT, inherent optical properties of tissue microstructures cause light to be scattered or absorbed. This lack of light transparency caused by tissue density, as well as noise and light artifacts, provides challenges when quantitatively analyzing the skin and when attempting to detect definitive layers of the epidermis and dermis. As such, typical image enhancement methods such as smoothing and sharpening do not provide much improvement; therefore, more sophisticated image processing algorithms with or without contrast agents or using other imaging modalities’ input will be utilized.^39^, ^50^, ^51^, ^52^, ^53^, ^54^, ^55^, ^56^, ^57^, ^58^, ^59^, ^60^, ^61^

**FIGURE 1:**
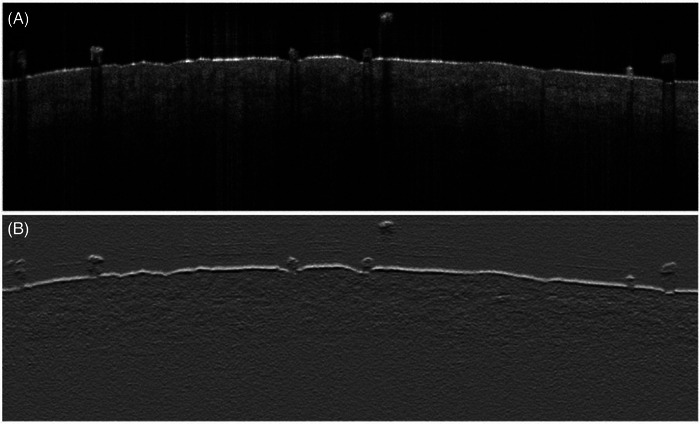
Attenuation coefficient (AC) map schematic. (a) Optical coherence tomography (OCT) image of melanoma and (b) AC map of the OCT image of the melanoma allows for improved visualization of the stratum corneum of the epidermis. The sizes of the AC map and original OCT image (a) are the same, demonstrating that our kernel shifts pixel by pixel.

To allow the algorithm to better detect the location of the stratum corneum of the epidermis on OCT, we translated the OCT image to an AC map (Figure 1a). The AC map is an image depicting calculated AC values of light attenuation as light penetrates into the skin through different ROIs. A convolution operation was used to generate the AC map by calculating numerous AC kernels on MATLAB. We used an AC kernel size of 5 × 5, which was applied to the entire OCT image. The AC map (Figure 1b) clearly demonstrates greater contrast of the surface of the skin, allowing us to use a simple high gradient detection algorithm to find surface layer.

**FIGURE 2:**
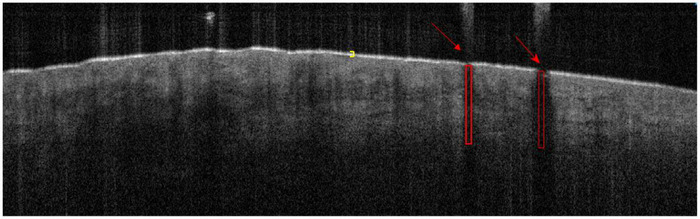
Invalid segments of the image that were omitted prior to attenuation coefficient (AC) analysis and mapping. Yellow bracket: stratum corneum aka skin entrance signal. Red arrow: hair shaft. Red rectangle: hair follicle.

To analyze OCT images for classification, we selected an ROI of 10 pixels in width and 150 pixels in height. A maximum of 78 ROIs per OCT image of a lesion were analyzed. Some OCT images had less than 78 ROIs selected due to manual deselection of areas of light artifact described later. The skin entrance signal correlates to the stratum corneum, which can be visualized as a hyperechoic line across the OCT image. Because including this bright signal intensity would positively skew the AC calculation and result in an inaccurate value, the skin entrance signal was omitted. Hence, we start the AC analysis immediately below the stratum corneum and end in the mid dermis where the OCT light source penetrates the skin deepest. Skin adnexa such as hair follicles and hair shafts (Figure 2), as well as light artifacts caused by dry skin flakes or dust, prevent light from adequately entering the skin, creating hypoechoic areas of the image. Because including these dark areas of the image would inaccurately and negatively skew AC values, these areas were manually deselected prior to analysis.

#### 2.3.3 Machine learning, Gabor wavelet transform, and support vector machine

**FIGURE 3:**
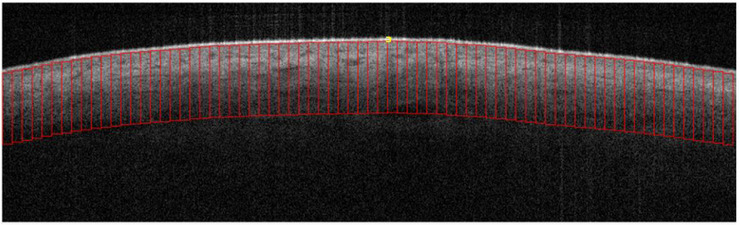
Regions of interest (ROIs) (red rectangle) selected from an optical coherence tomography (OCT) image. Each segment size is 10 pixels in width and 150 pixels in height. All ROIs begin below the stratum corneum (yellow bracket). Yellow bracket: stratum corneum aka skin entrance signal. Red vertical rectangle: ROI.

After the AC map was created and each of the ROI segments was identified and selected (Figure 3), each ROI from both the melanoma data set and benign nevi data set was fed into our machine learning algorithm. Segmentation, or the process of grouping the image's data into coherent sub‐sections based on features that were extracted, is a crucial step in inferring knowledge from an image.^62^ Segmentation is further divided into two categories: supervised and unsupervised.^62^ Unsupervised segmentation attempts to find common features between pixels and groups them naturally by relying on intensity and gradient image analysis.^63^ This method is useful when the borders of a tumor are well delineated.^62^ Because the borders of our target are not well delineated due to noise and light artifacts, we use a support vector machine (SVM), which is a supervised segmentation method.^64^, ^65^ Supervised segmentation relies on prior knowledge of the ground truth aided by human input where groups of pixels are pre‐labeled and trained as benign or malignant.^66^

For classification tasks, the supervised nature of SVM causes this algorithm to be highly dependent on feature extraction.^67^ Thus, the image must be translated into quantifiable numerical and textural data for computer‐based analysis. For our feature extraction, we determined wavelet transformation to be ideal. Wavelet transformation is widely used for frequency domain analysis and texture‐based feature analysis of an image.^68^ Frequency in an image processing is defined as change and diversity between pixels; for example, contrast between black and white has a high pixel value diversity and thus a high frequency.^69^, ^70^ Wavelet feature analysis allows for the localization of meaningful signals within an image in time and space and separates these signals from noise.^71^ We implement the Gabor wavelet filter, which is a group of wavelets, with each wavelet encompassing energy at specific frequency and orientation.^68^, ^72^ Textural and edge features can then be constructed from this data set of energy distributions.^68^, ^73^ The Gabor filter is a Gaussian kernel function governed by a sinusoidal component.^74^ The Gabor wavelet transformation formula^73^ is shown below, where *f* is the modulation frequency, and $\sigma_{x}$ and $\sigma_{y}$ represent Gaussian major and minor widths, respectively:

(1) $$g\left(x,y\right)=\frac{1}{2\pi \sigma_{x}\sigma_{y}}e^{\left(-\frac{x^{2}}{2\sigma_{x}^{2}}-\frac{y^{2}}{2\sigma_{y}^{2}}\right)+\left(j2\pi fx\right)}$$

**FIGURE 4:**
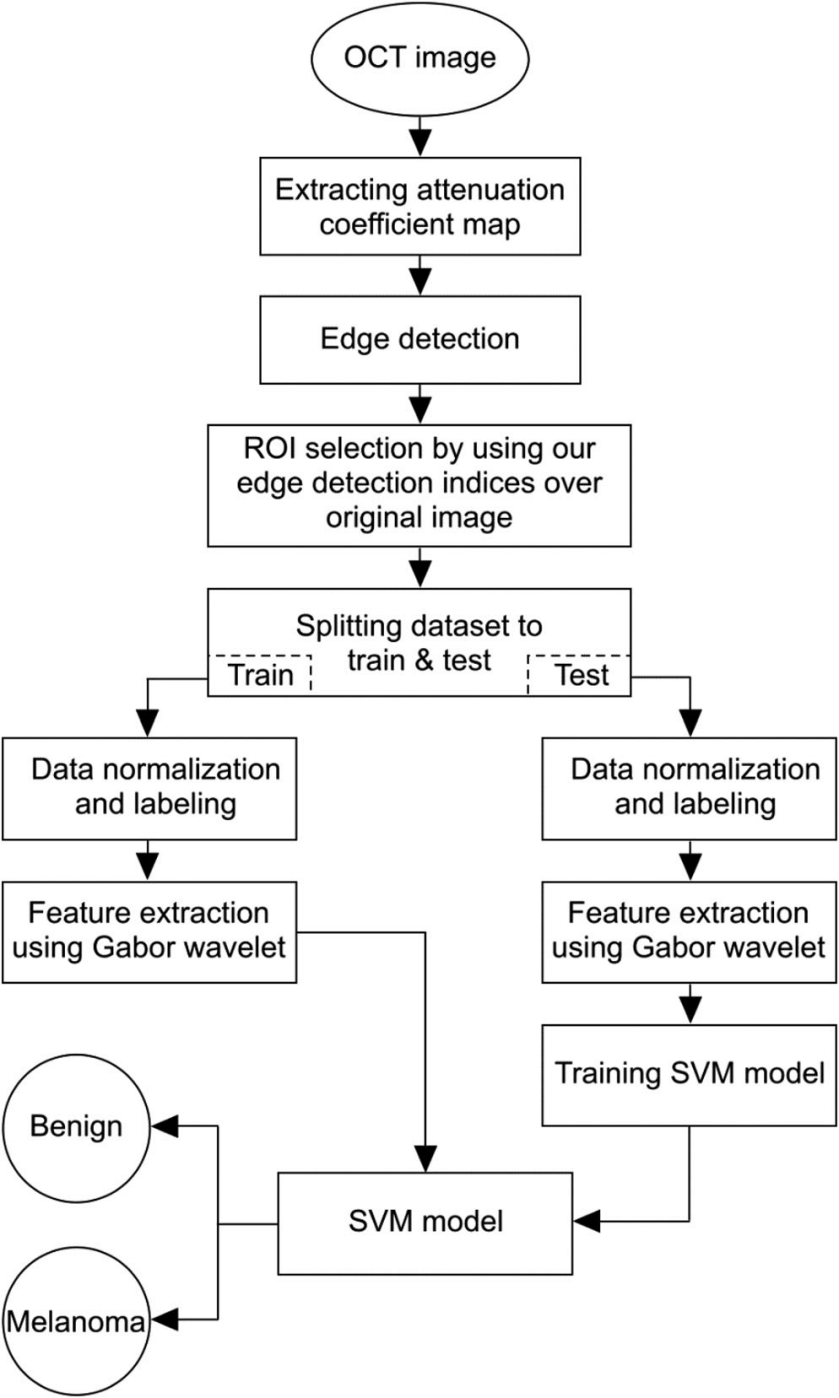
Classification algorithm flowchart.

We chose to use SVM because of its capability in creating the widest plane, or separation, between our two classes of benign and malignant. SVM is able to map points to other dimensions by use of nonlinear relationships for classification of data that is not linearly separable.^75^ In our methods, because our data are multidimensional (75 × 75 as opposed to typical 2D or 3D), we use a radial basis function kernel, where a real‐value function depends on the distance between the input and another fixed point such as the origin or elsewhere, called a center point.^76^ This allows for a multidimensional way to classify data with greater accuracy. For example, our OCT AC data are collected in an original space. SVM is able to map the data in hyperspace, or for example, the sine (etc.) of OCT ACs. This helps to classify data in a hyperspace, creating a hyperplane, which is positioned to optimally separate benign and malignant.^75^ The main goal of our method is to find the optimal hyperplane to classify data in the *n*‐dimension, which corresponds to the number of features extracted from our data. Our algorithm is detailed in the flowchart below (Figure 4).

## 3 RESULTS

### 3.1 OCT qualitative analysis

**FIGURE 5:**
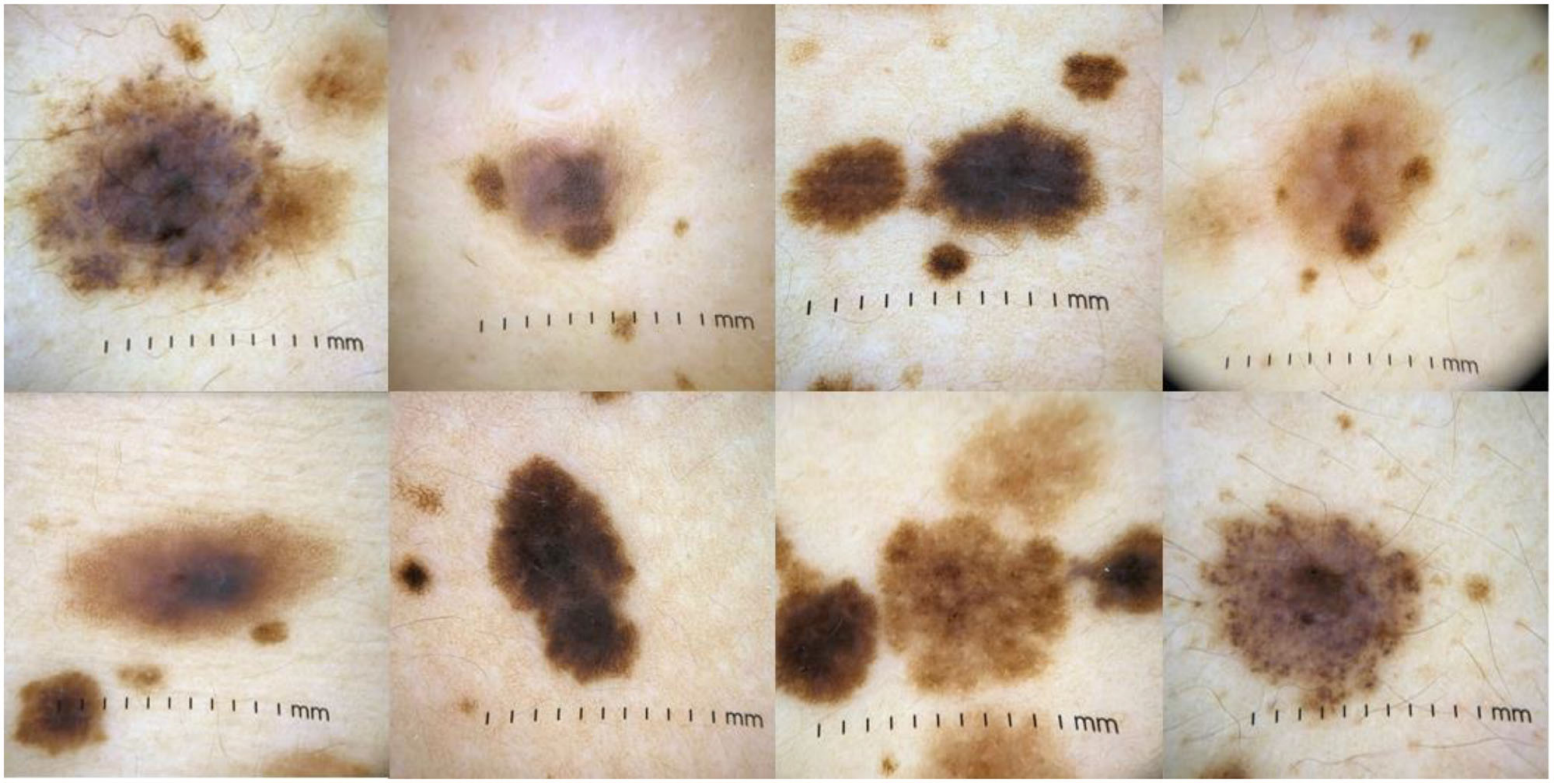
Dermoscopy photos of selected atypical nevi in phacomatosis pigmentokeratotica that may cause clinical suspicion of worrisome morphological atypia.

**FIGURE 6:**
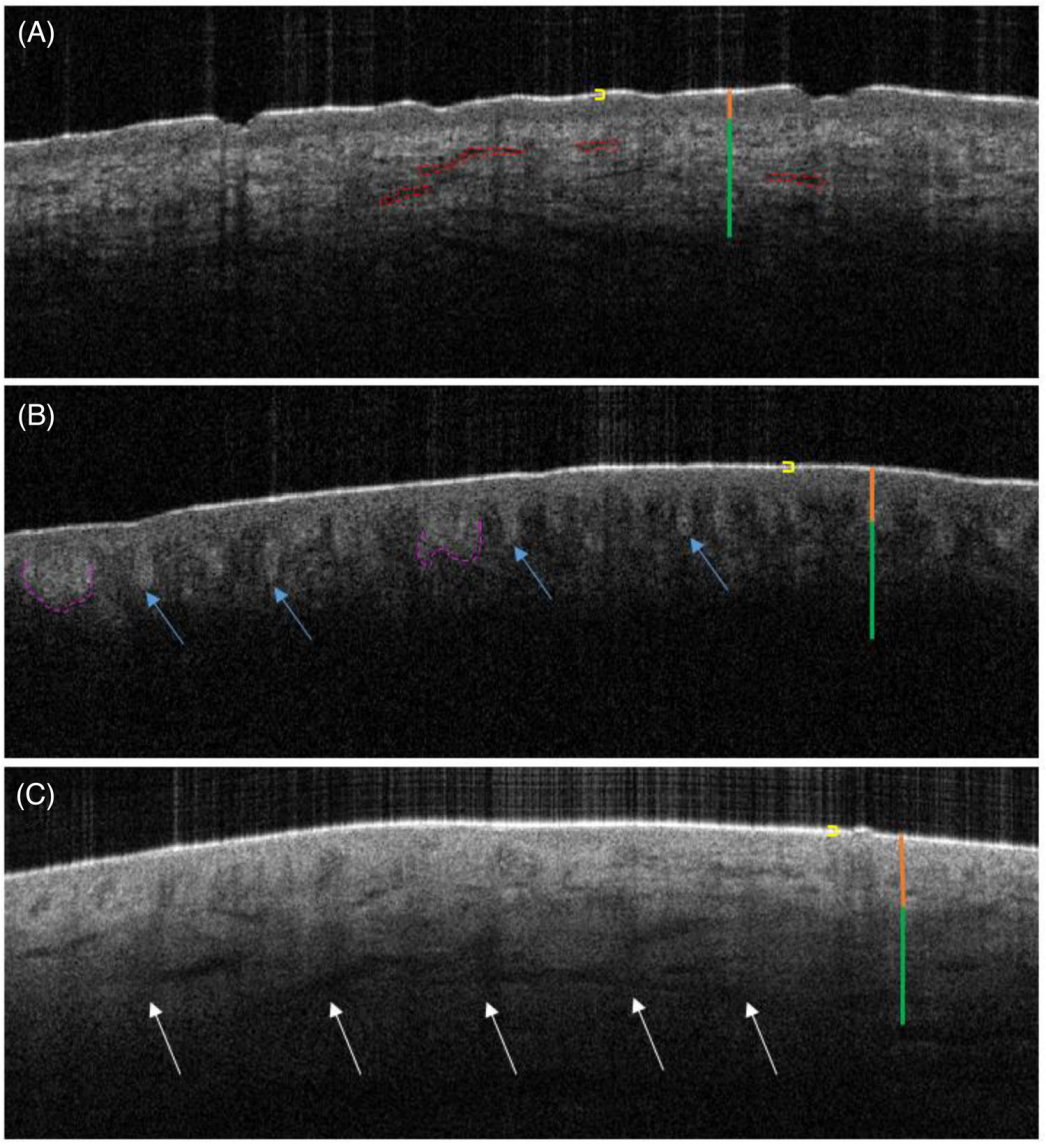
Optical coherence tomography (OCT) image comparisons of normal skin (a), nevus (b), and melanoma (c). (a) OCT image of normal skin. Neatly arranged skin layer architecture is observed. Yellow bracket: stratum corneum aka skin entrance signal. Orange line: epidermis. Green line: dermis with bright collagen fibers. Red dashed ovals: blood vessels. (b) OCT image of nevus. Blue arrows: elongated rete ridges. Pink dashed line: rete ridge fusion. Yellow bracket: stratum corneum aka skin entrance signal. Orange line: epidermis. Green line: dermis. (c) OCT image of melanoma in situ. Overall architectural disarray is notable, indicated by loss of a well‐delineated epidermis, dermis, and dermal–epidermal junction (DEJ). Dermal shadows (white arrows) are characteristic of melanoma on OCT. White arrows: vertical dermal shadows. Yellow bracket: stratum corneum aka skin entrance signal. Orange line: epidermis. Green line: dermis.

Dermoscopy images of examples of selected nevi are visualized in Figure 5. In OCT B‐scan images of normal skin (Figure 6a), clear delineations of layers of the skin can be visualized. The bright hyper‐reflective line correlates to the stratum corneum and the skin entrance signal.^77^ The more hypo‐reflective band immediately beneath correlates to the epidermis. The brighter signal intensity following the epidermis is the dermal–epidermal junction (DEJ), characterized by hyper‐reflective collagen bundles. Immediately underlying the DEJ is the papillary dermis, which further progresses downward into the reticular dermis at which point the signal is no longer present. Both the epidermis and dermis propagate, absorb, and scatter light more than the stratum corneum.^78^ Structures such as blood vessels and glands, which are indicated by hypo‐reflective dark lines or tubular structures, are seen throughout the dermis. Hair follicles are also visualized as indicated by vertical hypo‐reflective signals beginning within areas of the dermis and protruding outward toward the top of the skin.^77^ Overall tissue structure and layers of the skin are well organized and consistent throughout the image.

In pigmented lesions of PPK OCT (Figure 6b) B‐scan images, changes in skin layers are evident. Areas of atypical nevi with reticular network patterns visualized on dermoscopy correlate to elongated, broadened, irregular rete ridges accentuated by dense melanocytic nests at the tips on OCT.^79^ Contrasting with melanoma on OCT, nevi have a well‐delineated and preserved DEJ and a consistently undulating pattern of elongated rete ridges throughout the image. Rectification of rete ridges, or a flattened epidermis with hypo‐reflective area below, as well as fusion of rete pegs are also observed (Figure 6b).^80^

In melanoma OCT (Figure 6c) B‐scan images, architectural disarray is seen. Similar to thick atypical nevi, there is a lack of normal DEJ signal intensity, indicating effacement of the junction. Instead of linear, well‐demarcated sections of epidermis, DEJ, and dermis, the signal intensities are relatively homogenous throughout. Not only are melanoma rete ridges no longer as accentuated as benign nevi, but they are also shorter in length. Rete ridges, although rarely present in melanoma, display a significantly erratic profile compared to the consistent undulating pattern in benign nevi. Dense clusters of melanocytic nests and increased vascularity are seen. Differentiating features particular to melanoma OCT images are dermal shadows^81^ (Figure 6c) seen on OCT images of MIS and invasive melanomas and hyper‐reflective vertical icicle‐shaped structures reaching the reticular dermis for invasive melanomas.^79^, ^80^

### 3.2 OCT quantitative analysis

#### 3.2.1 Attenuation coefficient calculations

Initially, we attempted an unsupervised segmentation method by assessing only AC values of four benign nevi and four melanomas. We selected 20 ROI vertical segments (10 pixels in width × 150 pixels in depth), and divided each ROI into seven horizontal sections, separating the individual ROIs into epidermis (depth 1), DEJ (depth 2), superficial papillary dermis (depth 3), mid‐papillary dermis (depths 4 and 5), deep papillary dermis (depth 6), and superficial reticular dermis (depth 7) layers. Melanoma demonstrated lower AC values compared to benign nevi of PPK, especially at depths 1 (Figure 6), 3, and 7. For depths 2, 3, 5, and 6, 50% of the time, melanoma had higher AC values compared to benign nevi of PPK. In total, 18 out of 28 depth profiles (64.0%) of melanoma studied had lower AC values. For eight out of 28 depth profiles (28.0%), melanoma displayed higher AC values. For two out of 28 depth profiles (0.07%), PPK‐benign nevi ACs were comparable to melanoma. However, we found that these results had limited applicability and needed a wider scale, which initiated the use of our SVM supervised algorithm.

#### 3.2.2 Machine learning algorithm

A total of 86 OCT images were used to train and test the SVM model and algorithm. For the training phase, 74 OCT images were used (37 pigmented lesions in PPK and 37 melanoma). For the testing phase, 12 OCT images were used (six pigmented lesions in PPK and six melanoma). Each ROI (10 pixels in width × 150 pixels in depth) per OCT image was fed into the SVM feature extraction algorithm for image segmentation. A total of 9271 ROIs were extracted for the training set and 1809 ROIs were extracted for the testing set. During the test phase, a whole OCT image of either pigmented lesion in PPK or melanoma was used. The “Predicted Class Winner” indicates the class chosen with the highest probability based on ROI classification. Based on our results (Table 1), our algorithm was able to correctly classify whether or not the OCT image was truly benign or melanoma.

**TABLE 1:** Test case results for benign versus melanoma classification using support vector machine and Gabor transform wavelet feature.

Case number	True class	Number of roi per image	Number of predicted roi as benign	Number of predicted roi as melanoma	Predicted class winner
Case 1	Melanoma	150	33	117	Melanoma
Case 2	Melanoma	150	33	117	Melanoma
Case 3	Melanoma	147	32	115	Melanoma
Case 4	Melanoma	150	30	120	Melanoma
Case 5	Melanoma	150	40	110	Melanoma
Case 6	Melanoma	150	25	125	Melanoma
Case 7	Benign	152	103	49	Benign
Case 8	Benign	152	106	46	Benign
Case 9	Benign	152	127	25	Benign
Case 10	Benign	152	132	20	Benign
Case 11	Benign	152	137	15	Benign
Case 12	Benign	152	141	11	Benign

Abbreviation: ROI, region of interest.

For our binary classification, two classes were assigned: benign or melanoma. The confusion matrix (Table 2) represents our model's performance in identifying benign optical features in pigmented lesions in PPK. To evaluate the performance of the method, true positive (TP), true negative (TN), false positive (FP), and false negative (FN) values were calculated. TP indicates the number of lesions correctly classified. TN indicates the number of lesions that have been truly rejected. FP indicates the number of lesions that were incorrectly detected. FN indicates the number of lesions that were falsely rejected.

**TABLE 2:** Confusion matrix of our binary classification model.

Predicted			
Ground truth		Positive	Negative
	Positive	746	166
	Negative	193	704

*Note*: True positive: 746, true negative: 704, false positive: 193, and false negative: 166.

From the confusion matrix, precision and recall values were calculated from TP, TN, FP, and FN calculations. Precision quantifies the number of predicted positive classifications that truly belong to a positive class, also described as the reliability of the model. Precision reflects the ratio of the truly classified positive sample set to total number of positively classified samples. The precision formula is shown below:

(2) $$\mathrm{Precision}=\frac{\mathrm{True}\;\mathrm{positive}}{\mathrm{True}\;\mathrm{positive}+\mathrm{False}\;\mathrm{negative}}$$

Recall quantifies the number of predicted positive classifications made from the total number of positive examples from our set of data, also described as the ability of the model to classify *positive* samples.However, if the model detects all of the *positive* samples, recall will be 100% even if all *negative* samples were classified as *positive*, incorrectly. The recall formula is shown below:

(3) $$\mathrm{Recall}=\frac{\mathrm{True}\;\mathrm{positive}}{\mathrm{True}\;\mathrm{positive}+\mathrm{False}\;\mathrm{positive}}$$

Finally, the *F*1 score, which combines precision and recall, is calculated. The calculation for the *F*1 score is demonstrated in the formula below:

(4) $$F1=\frac{\mathrm{Precision}\times \mathrm{Recall}}{\mathrm{Precision}+\mathrm{Recall}}$$

For the testing phase in our SVM model for benign lesions, the precision calculation was 79%, with recall of 82% and *F*1 score of 81%. For the testing phase for melanoma lesions, the precision calculation was 81%, with recall of 78% and *F*1 score of 80%. The data are represented below (Table 3).

**TABLE 3:** Precision, recall, and *F*1 score for benign and melanoma classification using support vector machine and Gabor transform wavelet features.

	Precision	Recall	*F*1 score
Benign	79%	82%	81%
Melanoma	81%	78%	80%

## 4 DISCUSSION

PPK is a rare epidermal nevus syndrome characterized by the coexistence of nevus sebaceous and SLN with additional extracutaneous syndromic features that involve multiple organ systems.^2^, ^82^ Melanocytic proliferations within SLN may consist of many types of nevi which may include dysplastic, Spitz, and compound, oftentimes with generalized atypia. Such worrisome features may prompt clinicians to excise these lesions for suspicion of malignancy. However, for patients with PPK, biopsy of a large number of these melanocytic lesions may not be physically feasible, posing a challenge during evaluation. In a retrospective study analyzing biopsy rates of 18 485 biopsies of melanocytic proliferations, only 8.6% of them were diagnosed melanoma‐in‐situ or invasive melanoma.^83^ Consequently, non‐invasive imaging and other technologies have been developed in an attempt to decrease biopsy numbers; however, biopsies remain the gold standard. According to a Cochrane analysis, swept‐source OCT currently has a specificity of 61% in the discrimination of melanoma from nevi^40^ and additional studies have been able to increase the specificity to 80%.^31^ The aim of our study was to investigate if a post‐OCT imaging algorithm could confirm benign features of pigmented lesions in a patient with PPK, without necessitating invasive biopsy procedures. We have demonstrated that by implementing SVM, a high‐performance machine learning method, and Gabor wavelet transformation feature extraction, we have been able to confirm that the selected pigmented lesions imaged in PPK were benign. Furthermore, by implementing a backscattering equation followed by the calculation of ACs, we observed intrinsic differences in values between nevi in PPK when comparing them to melanoma.

Machine learning research in the scope of melanoma and nevi differentiation has previously been applied to dermoscopic and non‐dermoscopic gross images of the lesion with pixel‐by‐pixel analysis.^84^ SVM with Gabor wavelet transformation applications to melanoma versus nevi has been reported, but with analysis of histopathologic slide and dermoscopic images.^85^, ^86^ We are the first and only study thus far to apply SVM learning in conjunction with Gabor wavelet transformation to swept‐source OCT imaging of melanoma and benign nevi. Based on our training and testing set, we conclude that our algorithm was successfully able to predict benign diagnosis. Our results indicate a potential increased applicability of these methods to studies of a larger sample size.

Tissues have intrinsic optical properties that allow for the detection of changes in light scattering, absorption, and attenuation, which affect AC.^87^ By implementing the study of AC in our algorithm, we are able to discern why benign features can be discriminated from malignancy in OCT. Lower ACs indicate tissue features that are more absorbent and more transparent to light, and higher ACs indicate features that reflect or are more opaque to light.^87^ This is based on microstructure size, density, and shape, chromophore concentration, and cellularity order and disorder. During tumorigenesis, these optical properties change due to neoplastic disarray of normal cellular structure, allowing OCT to visualize these changes. OCT is sensitive in detecting the presence of neoplasm. However, specificity is low due to interrelated optical characteristics of tissue characteristics.^31^, ^88^ In this study, our methods of post‐image processing and analysis are able to identify and classify discrete patterns within OCT imaging, yielding *F*1 test scores of 81% and 80% for benign nevi and melanoma, respectively.

The microarchitecture of benign nevi is vastly different from melanoma, which contributes to the differences in AC values. Nevi may have higher AC values than melanoma due to less architectural effacement. Notably, epidermal hyperplasia, commonly seen in benign lesions, may contribute to higher AC values.^89^ Compound nevi are characterized by compact hyperkeratosis, orthokeratosis, and hypergranulosis.^90^, ^91^ Keratin in the epidermis exhibits bright light scattering, which decreases the absorption of light, thus increasing AC, as demonstrated in our results.^92^ Additionally, in Spitz nevi lesions, presence of Kamino bodies in the epidermis may cause an increase in AC values in pigmented lesions in PPK. Kamino bodies, which are absent in melanomas, are hyaline structures that trichrome stain similarly to collagen.^93^

Features that indicate malignancy, as seen in melanoma, include larger amounts of cytoplasm within melanocytes. Cytoplasm poorly scatters light, which causes a reduction in AC values compared to nevi.^94^ Melanoma epidermal ACs are 10 times lower than those of normal skin, indicating that the epidermis is more translucent compared to nevi. As such, light scattering is dependent on refractive index mismatch as the incident light beam moves through altered architecture.^81^, ^95^ Organelles within cells, such as the nucleus, also determine light scattering capabilities.^96^ Features that define melanoma include larger nuclear to cytoplasmic ratio, nuclear hyperchromasia, mitotic activity, and pleomorphism.^60^, ^97^ In melanoma, large nuclei with greater DNA content indicate rapid division of tumor cells, which display higher light scattering consistent with increased interception of light with increased smaller structures.^94^

Melanocytic maturation or lack thereof define benign nevi and melanoma, respectively. In benign nevi, melanocyte maturation is present, indicated by proliferative melanocytic nests beginning at the epidermis, trickling down to single file melanocytes toward the dermis. A lack of melanocyte maturity with clumps of disorganized melanocytic nests toward the dermis is a defining feature of melanoma.^98^, ^99^ These subtle differences may be observed in depth profiles where pigmented lesions ACs in PPK are higher than melanoma ACs. Because nevi have singular melanocytes entering the dermis, areas surrounding these dermal melanocytes may consist of lamellar and/or concentric dermal fibrosis, as demonstrated in compound dysplastic nevi.^100^ Compared to normal skin, smaller AC standard deviations may be observed in melanoma and atypical nevi due to greater tissue homogeneity in melanocyte content. For example, as the incident light enters melanoma or atypical nevi, it will encounter a maturation axis of consistent melanocytes. In contrast, in normal skin, layers of the skin are neatly organized with microstructures such as hair follicles, blood vessels, and sweat glands interweaved in their respective layers. As the light encounters different layers and different microstructures, signal intensities are observed on OCT, contributing to a wider range of AC values across each depth. Additionally, normal skin is sharply defined by the DEJ, which is characterized by collagen fibrils. Both melanoma and nevi lack a similar DEJ intensity, indicating the lack of organized band of collagen density and architectural disarray.^101^ All of these factors allow for meaningful pattern recognition by our SVM algorithm.

Swept‐source OCT allows for higher scan rate and less motion artifacts, allowing for improved image contrast.^101^, ^102^ However, limitations include light signal intensity decay, artifacts such as blood vessels, speckle noise produced in the OCT image, tissue or OCT probe motion, and blurring, all of which could affect pixel value and thus AC calculations.^101^ Bright signal intensities, such as high melanin pigment content, could reflect the light signal, thereby obstructing further light penetration to regions below. While resolution of OCT has not been able to display morphology of single cells, clear architectural changes can be seen. With implementation of post‐image processing by analyzing optical properties and AC values of melanoma, PPK‐atypical nevi, and normal skin, we can further characterize tissue features without biopsy. Often, however, to accurately diagnose dysplastic or Spitz nevi from melanoma, discrete and subtle histological findings must be seen.

Machine learning applications have limitations as well. Because machine learning is highly dependent on the data it is given, the quality of images must be clear and accurate. Poor‐quality images may cause machine learning to draw inaccurate conclusions and thus must be properly vetted.^103^ Additionally, machine learning does not take into consideration the effect of other physiological or pathological etiologies that might affect the presentation. Thus, machine learning and artificial intelligence in the scope of dermatologic applications should be used in combination with a physician's clinical interpretation to ensure a comprehensive diagnostic approach to melanocytic lesion management. In our study specifically, due to many options of feature extraction and selection methods, fine‐tuning these combinations may continue to optimize our method's specificity score.

## 5 CONCLUSION

Swept‐source OCT has been demonstrated to be a promising technological advancement in a step toward potentially decreasing biopsy numbers. While OCT lacks the ability to discriminate detail at the cellular level, the addition of post‐image processing and analysis by SVM machine learning, Gabor wavelet transformation, and AC has demonstrated the ability to confirm benign features in pigmented lesions in a patient with PPK. Studies with larger sample sizes must be explored to further investigate the utility of this non‐invasive post‐image analytical approach to pigmented lesions.

## CONFLICT OF INTEREST STATEMENT

The authors declare no conflicts of interest.

## Data Availability

The data that support the findings of this study are available from the corresponding author upon reasonable request.

## References

[1] Happle R , Hoffmann R , Restano L , Caputo R , Tadini G . Phacomatosis pigmentokeratotica: a melanocytic–epidermal twin nevus syndrome. Am J Med Genet. 1996;65(4):363‐365. 8923953 10.1002/(SICI)1096-8628(19961111)65:4<363::AID-AJMG27>3.0.CO;2-R

[2] Happle R . The group of epidermal nevus syndromes: Part I. Well defined phenotypes. J Am Acad Dermatol. 2010;63(1):1‐22. 20542174 10.1016/j.jaad.2010.01.017

[3] Tadini G , Restano L , Gonzáles‐Pérez R , et al. Phacomatosis pigmentokeratotica: report of new cases and further delineation of the syndrome. Arch Dermatol. 1998;134(3):333‐337. 9580120 10.1001/archderm.134.3.333

[4] Torchia D , Happle R . Phacomatosis spilosebacea: a new name for a distinctive binary genodermatosis. J Am Acad Dermatol. 2021. 10.1016/j.jaad.2020.12.082 33583608

[5] Schaffer JV , Orlow SJ , Lazova R , Bolognia JL . Speckled lentiginous nevus: within the spectrum of congenital melanocytic nevi. Arch Dermatol. 2001;137(2):172‐178. 11176689

[6] Kinsler VA , O'hare P , Bulstrode N , et al. Melanoma in congenital melanocytic naevi. British Journal of Dermatology. 2017; May 1;176(5):1131‐43 28078671 10.1111/bjd.15301 PMC5484991

[7] Da Forno P , Pringle J , Fletcher A , et al. BRAF, NRAS and HRAS mutations in spitzoid tumours and their possible pathogenetic significance. Br J Dermatol. 2009;161(2):364‐372. 19438459 10.1111/j.1365-2133.2009.09181.x

[8] Bastian BC , LeBoit PE , Pinkel D . Mutations and copy number increase of HRAS in Spitz nevi with distinctive histopathological features. Am J Pathol. 2000;157(3):967‐972. 10980135 10.1016/S0002-9440(10)64609-3 PMC1885704

[9] Groesser L , Herschberger E , Sagrera A , et al. Phacomatosis pigmentokeratotica is caused by a postzygotic HRAS mutation in a multipotent progenitor cell. J Invest Dermatol. 2013;133(8):1998‐2003. 23337891 10.1038/jid.2013.24

[10] Groesser L , Herschberger E , Ruetten A , et al. Postzygotic HRAS and KRAS mutations cause nevus sebaceous and Schimmelpenning syndrome. Nat Genet. 2012;44(7):783‐787. 22683711 10.1038/ng.2316

[11] Martínez‐Menchón T , Mahiques Santos L , Vilata Corell J , Febrer Bosch I , Fortea Baixauli JM . Phacomatosis pigmentokeratotica: a 20‐year follow‐up with malignant degeneration of both nevus components. Pediatr Dermatol. 2005;22(1):44‐47. 15660897 10.1111/j.1525-1470.2005.22110.x

[12] Fleming NH , Egbert BM , Kim J , Swetter SM . Reexamining the threshold for reexcision of histologically transected dysplastic nevi. JAMA Dermatol. 2016;152(12):1327‐1334. 27542070 10.1001/jamadermatol.2016.2869

[13] Hayek SA , Munoz A , Dove JT , et al. Hospital‐based study of compliance with NCCN guidelines and predictive factors of sentinel lymph node biopsy in the setting of thin melanoma using the national cancer database. Am Surg. 2018;84(5):672‐679. 29966567

[14] Friedman RJ , Rigel DS , Kopf AW . Early detection of malignant melanoma: the role of physician examination and self‐examination of the skin. CA Cancer J Clin. 1985;35(3):130‐151. 3921200 10.3322/canjclin.35.3.130

[15] Thomas L , Tranchand P , Berard F , Secchi T , Colin C , Moulin G . Semiological value of ABCDE criteria in the diagnosis of cutaneous pigmented tumors. Dermatology. 1998;197(1):11‐17. 9693179 10.1159/000017969

[16] Wilson RL , Yentzer BA , Isom SP , Feldman SR , Fleischer Jr AB . How good are US dermatologists at discriminating skin cancers? A number‐needed‐to‐treat analysis. J Dermatol Treat. 2012;23(1):65‐69. 10.3109/09546634.2010.512951 21756146

[17] Avanaki K , Andersen PE . Optical coherence tomography for melanoma detection. *New Technologies in Dermatological Science and Practice* . CRC Press; 2021:47‐58.

[18] Lukas B , May JR , tsoukas M , Avanaki K . Skin cancer detection using optical coherence tomography. Optical Spectroscopy and Imaging for Cancer Diagnostics: Fundamentals, Progress, and Challenges. World Scientific; 2023:473‐495.

[19] Rajabi‐Estarabadi A , Bittar JM , Zheng C , et al. Optical coherence tomography imaging of melanoma skin cancer. Lasers Med Sci. 2019;34:411‐420. 30539405 10.1007/s10103-018-2696-1

[20] Rajabi‐Estarabadi A , Bittar JM , Zheng C , et al. Features of Cutaneous Melanoma Using Dynamic Optical Coherence Tomography: A Case Report and Review of the Literature . In Journal of the american academy of dermatology, mosby‐elsevier, NY, USA, 2019, Vol. 81, No. 4, pp. AB212–AB212).

[21] Podoleanu AG . Optical coherence tomography. *J Microsc*. 2012;247(3):209‐219. 10.1111/j.1365-2818.2012.03619.x PMC3563006 22708800

[22] Welzel J , Lankenau E , Birngruber R , Engelhardt R . Optical coherence tomography of the human skin. J Am Acad Dermatol. 1997;37(6):958‐963. 9418764 10.1016/s0190-9622(97)70072-0

[23] Pratavieira S , Andrade CT , Salvio AG , Bagnato VS , Kurachi C . Optical imaging as auxiliary tool in skin cancer diagnosis. *Skin Cancers—Risk Factors, Prevention and Therapy*. 2011:159‐173.

[24] Tes D , Aber A , Zafar M , et al. Granular cell tumor imaging using optical coherence tomography. Biomed Eng Comput Biol. 2018;9:117959721879025. 10.1177/1179597218790250 PMC6088518 30116105

[25] Kwasniak LA , Garcia‐Zuazaga J . Basal cell carcinoma: evidence‐based medicine and review of treatment modalities. Int J Dermatol. 2011;50(6):645‐658. 21595656 10.1111/j.1365-4632.2010.04826.x

[26] Dubois A , Boccara AC . Full‐field optical coherence tomography. Optical Coherence Tomography: Technology and Applications. Springer; 2008:565‐591.

[27] Dubois A , Levecq O , Azimani H , et al. Line‐field confocal optical coherence tomography for high‐resolution noninvasive imaging of skin tumors. J Biomed Opt. 2018;23(10):106007‐106007. 10.1117/1.JBO.23.10.106007 30353716

[28] Braghiroli NF , Sugerik S , Freitas LAR , Oliviero M , Rabinovitz H . The skin through reflectance confocal microscopy—historical background, technical principles, and its correlation with histopathology. An Bras Dermatol. 2022;97(6):697‐703. doi:10.1016/j.abd.2021.10.010 36153173 PMC9582891

[29] Wang C , Guo L , Wang G , et al. In‐vivo imaging of melanoma with simultaneous dual‐wavelength acoustic‐resolution‐based photoacoustic/ultrasound microscopy. Appl Opt. 2021;60(13):3772‐3778. 33983310 10.1364/AO.412609

[30] Bohren CF , Huffman DR . Absorption and Scattering of Light by Small Particles. John Wiley & Sons; 2008.

[31] Turani Z , Fatemizadeh E , Blumetti T , et al. Optical radiomic signatures derived from optical coherence tomography images improve identification of melanoma. Cancer Res. 2019;79(8):2021‐2030. 30777852 10.1158/0008-5472.CAN-18-2791 PMC6836720

[32] Turani Z , Fatemizadeh E , Blumetti T , et al. Melanoma detection using quantitative analysis of optical coherence tomography images. In *Optical Interactions with Tissue and Cells XXXII*. SPIE; 2021; Vol. 11640, p. 116400T.

[33] Avanaki K , Andersen P . OCT radiomic features for differentiation of early malignant melanoma from benign nevus. Google Patents. 2020.

[34] Turani Z , Fatemizadeh E , Blumetti T , et al. Optical radiomic signatures derived from OCT images to improve identification of melanoma. In European Conference on Biomedical Optics, OPTICA 2019:11078_23. 10.1158/0008-5472.CAN-18-2791 PMC6836720 30777852

[35] Turani Z , Fatemizadeh E , Blumetti T , et al. Optical radiomic signatures derived from optical coherence tomography images improve identification of melanoma optical radiomic signatures for identification of melanoma. Cancer Res. 2019;79(8):2021‐2030. 30777852 10.1158/0008-5472.CAN-18-2791 PMC6836720

[36] Adabi S , Hosseinzadeh M , Noei S , et al. Universal in vivo textural model for human skin based on optical coherence tomograms. Sci Rep. 2017;7(1):1‐11. 29263332 10.1038/s41598-017-17398-8 PMC5738372

[37] Avanaki MR , Hojjat A , Podoleanu AG . Investigation of computer‐based skin cancer detection using optical coherence tomography. J Mod Opt. 2009;56(13):1536‐1544.

[38] Adabi S , Turani Z , Fatemizadeh E , Clayton A , Nasiriavanaki M . Optical coherence tomography technology and quality improvement methods for optical coherence tomography images of skin: a short review. Biomed Eng Comput Biol. 2017;8:117959721771347. 10.1177/1179597217713475 PMC5470862 28638245

[39] Hojjatoleslami A , Avanaki MR . OCT skin image enhancement through attenuation compensation. Appl Opt. 2012;51(21):4927‐4935. 22858930 10.1364/AO.51.004927

[40] di Ruffano LF , Dinnes J , Deeks JJ , et al. Optical coherence tomography for diagnosing skin cancer in adults. Cochrane Database Syst Rev. 2018;12(12):CD013189. 30521690 10.1002/14651858.CD013189 PMC6516952

[41] Perez‐Anker J , Puig S , Alos L , et al. Morphological evaluation of melanocytic lesions with three‐dimensional line‐field confocal optical coherence tomography: correlation with histopathology and reflectance confocal microscopy. A pilot study. Clin Exp Dermatol. 2022;47(12):2222‐2233. 35988042 10.1111/ced.15383

[42] Agozzino M , Moscarella E , Babino G , Caccavale S , Piccolo V , Argenziano G . The use of in vivo reflectance confocal microscopy for the diagnosis of melanoma. Expert Rev Anticancer Ther. 2019;19(5):413‐421. 30869538 10.1080/14737140.2019.1593829

[43] Levine A , Markowitz O . Introduction to reflectance confocal microscopy and its use in clinical practice. JAAD Case Rep. 2018;4(10):1014‐1023. doi:10.1016/j.jdcr.2018.09.019 30456275 PMC6232695

[44] Wang YJ , Wang JY , Wu YH . Application of cellular resolution full‐field optical coherence tomography in vivo for the diagnosis of skin tumours and inflammatory skin diseases: a pilot study. Dermatology. 2022;238(1):121‐131. 33946063 10.1159/000514686

[45] Schmitt AM . Principles and application of optical coherent tomography in dermatology. Dermatology. 2008;217(1):12. 18309239 10.1159/000118507

[46] Vermeer KA , Mo J , Weda JJ , Lemij HG , de Boer JF . Depth‐resolved model‐based reconstruction of attenuation coefficients in optical coherence tomography. Biomedical Opt Express. 2014;5(1):322‐337. 10.1364/BOE.5.000322 PMC3891343 24466497

[47] Liu J , Ding N , Yu Y , et al. Optimized depth‐resolved estimation to measure optical attenuation coefficients from optical coherence tomography and its application in cerebral damage determination. J Biomed Opt. 2019;24(3):1‐11. 10.1117/1.JBO.24.3.035002 PMC6975193 30834722

[48] Gong P , McLaughlin RA , Liew YM , Munro PR , Wood FM , Sampson DD . Assessment of human burn scars with optical coherence tomography by imaging the attenuation coefficient of tissue after vascular masking. J Biomed Opt. 2013;19(2):021111. 10.1117/1.JBO.19.2.021111 24192908

[49] Xu C , Schmitt JM , Carlier SG , Virmani R . Characterization of atherosclerosis plaques by measuring both backscattering and attenuation coefficients in optical coherence tomography. J Biomed Opt. 2008;13(3):034003. 18601548 10.1117/1.2927464

[50] Eybposh MH , Turani Z , Mehregan D , Nasiriavanaki M . Cluster‐based filtering framework for speckle reduction in OCT images. Biomedical Opt Express. 2018;9(12):6359‐6373. 10.1364/BOE.9.006359 PMC6490990 31065434

[51] Turani Z , Fatemizadeh E , Daveluy S , Mehregan D , Manwar R , Avanaki M . Compensation of refractive index variation in optical coherence tomography images. InOptical Coherence Tomography and Coherence Domain Optical Methods in Biomedicine XXIII, SPIE, 2019, Vol. 10867, pp. 123‐129.

[52] Hojjatoleslami S , Avanaki M , Podoleanu AG . Image quality improvement in optical coherence tomography using Lucy–Richardson deconvolution algorithm. Appl Opt. 2013;52(23):5663‐5670. 23938416 10.1364/AO.52.005663

[53] Adabi S , Rashedi E , Clayton A , et al. Learnable despeckling framework for optical coherence tomography images. J Biomed Opt. 2018;23(1):1‐12. 10.1117/1.JBO.23.1.016013 29368458

[54] Xu Q , Jalilian E , Fakhoury JW , et al. Monitoring the topical delivery of ultrasmall gold nanoparticles using optical coherence tomography. Skin Res Technol. 2020;26(2):263‐268. 31556193 10.1111/srt.12789 PMC7058498

[55] Adabi S , Fotouhi A , Xu Q , et al. An overview of methods to mitigate artifacts in optical coherence tomography imaging of the skin. Skin Res Technol. 2018;24(2):265‐273. 29143429 10.1111/srt.12423

[56] Kratkiewicz K , Manwar R , Rajabi‐Estarabadi A , et al. Photoacoustic/ultrasound/optical coherence tomography evaluation of melanoma lesion and healthy skin in a swine model. Sensors. 2019;19(12):2815. 31238540 10.3390/s19122815 PMC6630987

[57] Panchal R , Horton L , Poozesh P , Baqersad J , Nasiriavanaki M . Vibration analysis of healthy skin: toward a noninvasive skin diagnosis methodology. J Biomed Opt. 2019;24(1):1‐11. 10.1117/1.JBO.24.1.015001 PMC6985698 30666853

[58] Avanaki MR , Hojjatoleslami A . Skin layer detection of optical coherence tomography images. Optik. 2013;124(22):5665‐5668.

[59] Turani Z , Fatemizadeh E , Xu Q , Daveluy S , Mehregan D , Nasiri Avanaki MR . Refractive index correction in optical coherence tomography images of multilayer tissues. J Biomed Opt. 2018;23(7):1‐4. 10.1117/1.JBO.23.7.070501 29992800

[60] Hessler M , Jalilian E , Xu Q , et al. Melanoma biomarkers and their potential application for in vivo diagnostic imaging modalities. Int J Mol Sci. 2020;21(24):9583. 33339193 10.3390/ijms21249583 PMC7765677

[61] Jalilian E , Xu Q , Horton L , et al. Contrast‐enhanced optical coherence tomography for melanoma detection: an in vitro study. J Biophoton. 2020;13(5):e201960097. 10.1002/jbio.201960097 32072773

[62] Seo H , Badiei Khuzani M , Vasudevan V , et al. Machine learning techniques for biomedical image segmentation: an overview of technical aspects and introduction to state‐of‐art applications. Med Phys. 2020;47(5):e148‐e167. 32418337 10.1002/mp.13649 PMC7338207

[63] Nadif M , Role F . Unsupervised and self‐supervised deep learning approaches for biomedical text mining. Briefings Bioinf. 2021;22(2):1592‐1603. 10.1093/bib/bbab016 33569575

[64] Li J , Allinson N , Tao D , Li X . Multitraining support vector machine for image retrieval. IEEE Trans Image Process. 2006;15(11):3597‐3601. 17076417 10.1109/tip.2006.881938

[65] Ben‐Hur A , Weston J . A user's guide to support vector machines. *Data Mining Techniques for the Life Sciences* . Springer; 2010:223‐239. 10.1007/978-1-60327-241-4_13 20221922

[66] Sidey‐Gibbons JA , Sidey‐Gibbons CJ . Machine learning in medicine: a practical introduction. BMC Med Res Method. 2019;19(1):1‐18. 10.1186/s12874-019-0681-4 PMC6425557 30890124

[67] Lim TS , Loh WY , Shih YS . A comparison of prediction accuracy, complexity, and training time of thirty‐three old and new classification algorithms. Mach Learn. 2000;40(3):203‐228.

[68] Singh SM , Rajkumar R , Hemachandran K . Comparative study on content based image retrieval based on Gabor texture features at different scales of frequency and orientations. Int J Comp Appl. 2013;78(7):1‐7.

[69] Broughton SA , Bryan K . Discrete Fourier Analysis and Wavelets: Applications to Signal and Image Processing. John Wiley & Sons; 2018.

[70] Peli EJJA . Contrast in complex images. J Opt Soc Am A. 1990;7(10):2032‐2040. 2231113 10.1364/josaa.7.002032

[71] Wahab MF , O'Haver TC . Wavelet transforms in separation science for denoising and peak overlap detection. J Sep Sci. 2020;43(9‐10):1998‐2010. 32108426 10.1002/jssc.202000013

[72] Manjunath BS , Ma WY . Texture features for browsing and retrieval of image data. IEEE Trans Pattern Anal Mach Intell. 1996;18(8):837‐842.

[73] Vinay A , Shekhar VS , Murthy KB , Natarajan S . Face recognition using Gabor wavelet features with PCA and KPCA—a comparative study. Proc Comp Sci. 2015;57:650‐659.

[74] Sejdić E , Djurović I , Jiang J . Time–frequency feature representation using energy concentration: an overview of recent advances. Digital Signal Process. 2009;19(1):153‐183.

[75] Erickson BJ , Korfiatis P , Akkus Z , Kline TL . Machine learning for medical imaging. Radiographics. 2017;37(2):505. 28212054 10.1148/rg.2017160130 PMC5375621

[76] Guenther N , Schonlau M. Support vector machines. The Stata Journal. 2016; Dec;16(4):917‐37.

[77] Meyers AD . Optical Detection of Cancer. World Scientific; 2012.

[78] Krishnaswamy A , Baranoski GV . A biophysically‐based spectral model of light interaction with human skin. Wiley Online Library. 2004;23(3):331‐340.

[79] Gambichler T , Regeniter P , Bechara FG , et al. Characterization of benign and malignant melanocytic skin lesions using optical coherence tomography in vivo. J Am Acad Dermatol. 2007;57(4):629‐637. 17610989 10.1016/j.jaad.2007.05.029

[80] Blumetti TCMP , Cohen MP , Gomes EE , et al. Optical coherence tomography (OCT) features of nevi and melanomas and their association with intraepidermal or dermal involvement: a pilot study. J Am Acad Dermatol. 2015;73(2):315‐317. 26183975 10.1016/j.jaad.2015.05.009

[81] Boone M , Suppa M , Dhaenens F , et al. In vivo assessment of optical properties of melanocytic skin lesions and differentiation of melanoma from non‐malignant lesions by high‐definition optical coherence tomography. Arch Dermatol Res. 2016;308(1):7‐20. 26563265 10.1007/s00403-015-1608-5 PMC4713458

[82] Lee J , Benavides J , Manwar R , et al. Noninvasive imaging exploration of phacomatosis pigmentokeratotica using high‐frequency ultrasound and optical coherence tomography: can biopsy of PPK patients be avoided? Skin Res Technol. 2023;29(4):e13279. 37113090 10.1111/srt.13279 PMC10234170

[83] Lott JP , Boudreau DM , Barnhill RL , et al. Population‐based analysis of histologically confirmed melanocytic proliferations using natural language processing. JAMA Dermatol. 2018;154(1):24‐29. 29094145 10.1001/jamadermatol.2017.4060 PMC5833584

[84] Jafari MH , Nasr‐Esfahani E , Karimi N , Soroushmehr SMR , Samavi S , Najarian K . Extraction of skin lesions from non‐dermoscopic images for surgical excision of melanoma. Int J Comput Assist Radiol Surg. 2017;12(6):1021‐1030. 28342106 10.1007/s11548-017-1567-8

[85] Mahmoud MKA , Al‐Jumaily A . A Hybrid System for Skin Lesion Detection: Based on Gabor Wavelet and Support Vector Machine . CRC Press; 2014:14‐16.

[86] Bakheet S , Al‐Hamadi A . Computer‐aided diagnosis of malignant melanoma using Gabor‐based entropic features and multilevel neural networks. Diagnostics. 2020;10(10):822. 33066517 10.3390/diagnostics10100822 PMC7602255

[87] Drexler W , Fujimoto JG . Optical Coherence Tomography: Technology and Applications. Vol 2. Springer; 2015.

[88] Turchin IV , Sergeeva EA , Dolin LS , Shakhova NM , Richards‐Kortum R . Novel algorithm of processing optical coherence tomography images for differentiation of biological tissue pathologies. J Biomed Opt. 2005;10(6):064024. 16409089 10.1117/1.2137670

[89] Piris A , Mihm MC . Progress in melanoma histopathology and diagnosis. Hematol Oncol Clin North Am. 2009;23(3):467‐480. 19464597 10.1016/j.hoc.2009.03.012

[90] Horenstein MG , Prieto VG , Burchette Jr JL , Shea CR . Keratotic melanocytic nevus: a clinicopathologic and immunohistochemical study. J Cutan Pathol. 2000;27(7):344‐350. 10917161 10.1034/j.1600-0560.2000.027007344.x

[91] Verardino GC , Rochael MC . Spitz nevi in the classic histopathological pattern‐lamb in wolfs clothing. An Bras Dermatol. 2015;90:91‐95. 10.1590/abd1806-4841.20153310 PMC4323702 25672303

[92] Olsen J , Themstrup L , Jemec GBE . Optical coherence tomography in dermatology. G Ital Dermatol Venereol. 2015;150(5):603‐615. 26129683

[93] Venkatesh D , Smitha T . Kamino bodies. J Oral Maxillofac Pathol. 2019;23(1):17. 10.4103/jomfp.JOMFP_84_19 PMC6503810 31110411

[94] Mourant JR , Canpolat M , Brocker C , et al. Light scattering from cells: the contribution of the nucleus and the effects of proliferative status. J Biomed Opt. 2000;5(2):131‐137. 10938776 10.1117/1.429979

[95] Yi J , Backman V . Imaging a full set of optical scattering properties of biological tissue by inverse spectroscopic optical coherence tomography. Opt Lett. 2012;37(21):4443‐4445. 23114323 10.1364/OL.37.004443 PMC3640644

[96] Mourant JR , Freyer JP , Hielscher AH , Eick AA , Shen D , Johnson TM . Mechanisms of light scattering from biological cells relevant to noninvasive optical‐tissue diagnostics. Appl Opt. 1998;37(16):3586‐3593. 18273328 10.1364/ao.37.003586

[97] Peters MS , Goellner JR . Spitz naevi and malignant melanomas of childhood and adolescence. Histopathology. 1986;10(12):1289‐1302. 3817764 10.1111/j.1365-2559.1986.tb02572.x

[98] Crotty KA , Scolyer RA , Li LXL , Palmer AA , Wang L , McCarthy SW . Spitz naevus versus Spitzoid melanoma: when and how can they be distinguished? Pathology (Phila). 2002;34(1):6‐12. 10.1080/00313020120111212-1 11902448

[99] Menge TD , Pellacani G . Advances in noninvasive imaging of melanaoma. Semin Cutan Med Surg. 2016;35(1):18‐24. 26963113 10.12788/j.sder.2016.003

[100] Toussaint S , Kamino H . Dysplastic changes in different types of melanocytic nevi. A unifying concept. J Cutan Pathol. 1999;26(2):84‐90. 10082398 10.1111/j.1600-0560.1999.tb01807.x

[101] Adabi S , Turani Z , Fatemizadeh E , Clayton A , Nasiriavanaki M . Optical coherence tomography technology and quality improvement methods for optical coherence tomography images of skin: a short review. Biomed Eng Comput Biol. 2017;8:117959721771347. 10.1177/1179597217713475 PMC5470862 28638245

[102] O'leary S , Fotouhi A , Turk D , et al. OCT image atlas of healthy skin on sun‐exposed areas. Skin Res Technol. 2018;24(4):570‐586. 29575271 10.1111/srt.12468

[103] Nichols JA , Herbert Chan HW , Baker MAB . Machine learning: applications of artificial intelligence to imaging and diagnosis. Biophys Rev. 2019;11(1):111‐118. 30182201 10.1007/s12551-018-0449-9 PMC6381354

